# A study on the association between psychological capital and dyadic coping among lung cancer patients and their caregivers: an actor–partner interdependence model approach

**DOI:** 10.3389/fpsyg.2026.1940535

**Published:** 2026-09-17

**Authors:** Yan Li, Liu He, Vimala Ramoo, Xingling Zeng, Gek Mui Lim, Juan Du, Yuhong Zhang, Xiaoya Liu, Xinjing Xiang

**Affiliations:** 1 Faculty of Health Sciences, Nursing, and Education, MAHSA University, Jenjarom, Selangor, Malaysia; 2 The Second Affiliated Hospital of Chengdu Medical College, Nuclear Industry 416 Hospital, Chengdu, China; 3 Faculty of Medical and Life Sciences, Sunway University, Jalan University, Bandar Sunway, Selangor, Malaysia; 4 School of Nursing, Jiangsu Food & Pharmaceutical Science College, Huai’an, Jiangsu, China

**Keywords:** actor–partner interdependence model, dyadic coping, lung cancer, patient–caregiver dyads, psychological capital, psycho-oncology

## Abstract

**Background:**

Lung cancer imposes heavy physical and psychological burdens on both patients and their caregivers. Increasing evidence suggests that dyadic coping—the joint process through which patient–caregiver pairs manage illness-related stress—plays a crucial role in emotional adjustment and relationship quality. Meanwhile, psychological capital (PsyCap), comprising optimism, hope, resilience, and self-efficacy, has been recognized as a key positive psychological resource that enhances adaptation to cancer-related challenges. However, research integrating these two constructs within the lung cancer context remains limited.

**Methods:**

A cross-sectional study was conducted among 225 lung cancer patient–caregiver dyads recruited from hospitals in Chengdu between January and September 2025. Participants completed the Positive Psychological Capital Questionnaire (PPQ) and the Dyadic Coping Inventory (DCI). Pearson correlation analysis was used to examine bivariate associations, and the Actor–Partner Interdependence Model (APIM) was applied using AMOS 23.0 to explore actor and partner effects between PsyCap and dyadic coping.

**Results:**

Significant positive correlations were found between patients’ and their caregivers’ PsyCap and dyadic coping (*p* < 0.01). APIM analysis revealed that both patients’ (*β* = 0.17, *p* = 0.013) and caregivers’ (*β* = 0.23, *p* < 0.001) PsyCap significantly predicted their own dyadic coping (actor effects). The caregiver’s partner effect on the patient’s coping was significant (*β* = 0.14, *p* = 0.037), while the reverse effect was not significant. The dyad conformed to a mixed interdependence pattern (*k* = 0.633, 95% CI [0.125, 1.169]).

**Conclusion:**

Both patients’ and caregivers’ PsyCap were positively associated with dyadic coping. In the APIM, caregivers’ PsyCap showed a significant partner effect on patients’ dyadic coping, whereas the reverse partner path was not significant. Given the cross-sectional design, these findings should be interpreted as associations rather than causal effects. Longitudinal and intervention studies are needed to determine whether enhancing PsyCap can improve dyadic coping and related psychosocial outcomes in lung cancer dyads.

## 1 Introduction

Lung cancer is one of the most common cancers and the leading cause of cancer-related death worldwide (Thandra et al., 2021; Siegel et al., 2023). Due to its low early diagnostic rate, short survival period, lengthy treatment cycles, high recurrence rates, and substantial economic burden, patients with lung cancer experience heavier symptom distress and psychological pressure compared to patients with other cancers (Morrison et al., 2017). These challenges often lead to negative emotional states, immune dysfunction, increased mortality, and a decline in quality of life (Tu et al., 2021; Yu et al., 2022).

According to the Systemic Transactional Model (STM) proposed by Bodenmann and colleagues, individuals’ psychological states and behaviors can influence their partners through bidirectional feedback mechanisms of emotion, cognition, and behavior, leading to either adaptive or maladaptive relationship outcomes (Zemp et al., 2017). Dyadic coping refers to the process through which intimate partners mutually support each other and make joint decisions when facing chronic life events or illness (An et al., 2019). Existing research on dyadic coping has primarily focused on spousal relationships, although a few studies have extended this concept to include patient–caregiver dyads beyond spouses. A higher level of dyadic coping indicates stronger mutual support behaviors, which are beneficial for cancer management (von Heymann-Horan et al., 2019). Studies have shown that lung cancer caregivers bear dual responsibilities—ensuring patients’ daily functioning and improving their quality of life—while also experiencing substantial psychological stress (Palos et al., 2011). Clearly, cancer impacts not only patients’ health but also caregivers’ wellbeing, so coping with lung cancer should involve the patient–caregiver dyad rather than focus only on individuals.

With the emergence of positive psychology, Luthans and Youssef (Luthans and Youssef, 2004) proposed the concept of positive psychological capital (PsyCap), consisting of optimism, hope, resilience, and self-efficacy. In cancer patients, high PsyCap helps them adjust to illness-related challenges, reduce anxiety and depression, and improve treatment adherence (Harms et al., 2019). Likewise, caregivers with higher PsyCap are less likely to experience excessive burden and anticipatory grief. Thus, PsyCap plays a crucial role in maintaining the psychological health of both lung cancer patients and their caregivers (Sun et al., 2024).

The relationship between psychological capital and dyadic coping remains a relatively new field. While some studies have examined this relationship in breast cancer couples, limited research has explored the correlation and interaction mechanisms between these constructs in lung cancer—a malignancy characterized by rapid onset, heavy symptom burden—which makes dyadic coping particularly critical for this population. This study employed the Actor–Partner Interdependence Model (APIM) to investigate the interactive effects of psychological capital and dyadic coping among lung cancer patients and their caregivers, aiming to provide new insights for family-based psychosocial interventions.

## 2 Materials and methods

### 2.1 Participants

This study adopted a cross-sectional design and used convenience sampling. A total of 225 dyads of lung cancer patients and their primary caregivers were recruited from one tertiary hospital in Chengdu, Sichuan Province, China, between January and September 2025. This study was conducted in accordance with the Declaration of Helsinki and was approved by the Medical Ethics Committee of the Nuclear Industry 416 Hospital (Approval No.: YJ-2025-044-01). Written informed consent to participate in this study was provided by the patients and their primary caregivers.

#### 2.1.1 Inclusion and exclusion criteria

Inclusion criteria: Patients: (i) diagnosed with lung cancer via pathological or cytological examination; (ii) aware of their diagnosis; (iii) aged ≥ 18; (iv) in stable condition with clear consciousness and able to communicate effectively. Caregivers: (i) provided consistent care for at least 72 h; (ii) aged ≥ 18 years; (iii) with clear consciousness and communication ability.

Exclusion criteria: Patients: (i) diagnosed with other malignancies or life-threatening diseases; (ii) expected survival <3 months or extremely poor physical condition; (iii) with visual, hearing, or cognitive impairment; (iv) currently participating in other psychosocial studies or receiving psychiatric/psychological treatment. Caregivers: (i) with visual, hearing, or cognitive impairment; (ii) participating in other psychosocial studies or receiving psychiatric/psychological treatment.

#### 2.1.2 Sample size calculation

Sample size was calculated using G*Power software. According to the requirements of the APIM, assuming a medium effect size (*f*^2^ = 0.15), *α* = 0.05, and power (1 − *β*) = 0.90, the minimum required sample size was 202 dyads. Given an anticipated minimum invalidity rate of 10% for both patient and caregiver questionnaires, we conservatively inflated the sample size to ensure sufficient valid responses. Ultimately, 252 dyadic questionnaires were administered.

### 2.2 Research instruments

#### 2.2.1 Demographic information questionnaire

Designed by the research team, this collected participants’ demographic and disease-related data.

#### 2.2.2 Positive Psychological Capital Questionnaire (PPQ)

Developed by Zhang Kuo et al., the PPQ includes four dimensions—optimism, hope, resilience, and self-efficacy—rated on a 7-point Likert scale (total score range: 26–182) (Zhang et al., 2010). Higher scores indicate stronger PsyCap. In this study, Cronbach’s *α* coefficients for patients and caregivers were 0.752 and 0.900, respectively. Regarding the four subscales, Cronbach’s *α* coefficients (computed separately for each group) ranged from 0.604 to 0.804.

#### 2.2.3 Dyadic Coping Inventory (DCI)

Originally developed by Bodenmann and later cross-culturally adapted by Xu et al., the DCI uses a 5-point Likert scale (total score range: 35–175), where higher scores indicate better dyadic coping (Xu et al., 2016). The DCI was initially designed for couples. However, given that many cancer caregivers are non-spousal (e.g., parents, children, or other relatives), the present study adapted item wording to include these caregivers following the approach of von Heymann-Horan et al. (2019). In this study, Cronbach’s *α* coefficients were 0.804 for patients and 0.791 for caregivers. Regarding the five subscales, Cronbach’s *α* coefficients (computed separately for each group) ranged from 0.628 to 0.893.

### 2.3 Data collection

After obtaining informed consent, trained researchers guided patients and caregivers to independently complete the questionnaires in separate rooms (wards or conference rooms) to avoid mutual influence. All questionnaires were immediately checked for completeness to ensure data integrity. Researchers maintained effective communication with participants throughout, clarified any ambiguities in real time, and ensured strict confidentiality of all collected information.

### 2.4 Statistical analysis

All data were analyzed using SPSS 27.0 and AMOS 23.0. Continuous variables with a normal distribution were expressed as mean ± standard deviation (*M* ± SD) and compared using paired-samples *t*-tests. Categorical variables were presented as frequency and percentage [*n*(%)], with comparisons made using the *χ*^2^ test. Pearson correlation analysis was used to examine associations between patients’ and caregivers’ PsyCap and dyadic coping. The APIM was constructed in AMOS to evaluate actor and partner effects between patients’ and caregivers’ PsyCap and dyadic coping. To examine the robustness of the APIM findings, an additional covariate-adjusted APIM was estimated by including patient cancer stage and comorbidity burden as covariates. Harman’s single-factor test was conducted separately for patients and caregivers to assess potential common method variance, with a first-factor variance contribution below 40% considered indicative of no dominant common factor. A two-tailed *p* < 0.05 was considered statistically significant.

## 3 Results

### 3.1 General information of lung cancer patients and their caregivers

We collected 225 valid questionnaires (effective response rate 89.29%). The demographic characteristics of the patients are shown in Table 1. Among the 225 caregivers, spouses accounted for 71.11%, followed by children (20.89%). The caregivers included 100 men (44.44%) and 125 women (55.56%). Their ages were: ≤50 years (23.56%), 51–60 years (30.22%), 61–70 years (35.56%), and ≥71 years (10.67%). Regarding education, 35.11% had primary education or below; 34.67% had junior high school; 17.78% had high school or technical secondary school; 12.44% had college or above. The caregiving duration was <1 year for 25.33%, 1–3 years for 37.78%, and >3 years for 36.89%. Additionally, 75.11% of caregivers balanced caregiving with work, and 67.11% had chronic diseases themselves.

**Table 1:** Demographic information of lung cancer patients.

Indicators	*N* (%)	Indicators	*N* (%)
Sex		Health insurance status	
Male	157 (69.78)	Urban resident basic medical insurance	59 (26.22)
Female	68 (30.22)	New rural cooperative medical insurance	37 (16.44)
Age (years)		Urban employee basic medical insurance	121 (53.78)
≤50	16 (7.11)	Self-paying	8 (3.56)
51–60	57 (25.33)	Living situation	
61–70	80 (35.56)	Living only with spouse	88 (39.11)
≥71	72 (32.00)	Living with children and (or) parents	24 (10.67)
Marital status		Living with spouse, children and (or) parents	102 (45.33)
Unmarried	3 (1.33)	Living alone or with others	11 (4.89)
Married	197 (87.56)	Smoking status	
Divorced	8 (3.56)	Never smoked	139 (61.78)
Widowed	17 (7.56)	Former smoker	55 (24.44)
Educational level		Current smoker	31 (13.78)
Primary school and below	84 (37.33)	Number of comorbid chronic diseases (types)	
Middle school	98 (43.56)	0	78 (34.67)
High school/vocational school	26 (11.56)	1–2	106 (47.11)
Junior college or higher	17 (7.56)	≥3	41 (18.22)
Number of children (*n*)		Hospitalizations due to lung cancer (times)	
0	11 (4.89)	1–2	86 (38.22)
1–2	175 (77.78)	≥3	139 (61.78)
≥3	39 (17.33)	Pathological staging	
Residential area		Stage 1–2	22 (9.78)
Rural	90 (40.00)	Stage 3	79 (35.11)
Urban	135 (60.00)	Stage 4	124 (55.11)
Monthly family income/yuan		Frequency of social outings	
≤3,000	78 (34.67)	Less than once per week	124 (55.11)
3,001–4,999	114 (50.67)	1–2 times per week	87 (38.67)
≥5,000	33 (14.67)	Three or more times per week	14 (6.22)

### 3.2 Status and comparison of psychological capital and dyadic coping

Harman’s single-factor test was conducted separately for patients and caregivers to assess potential common method variance, with a first-factor variance contribution below 40% considered indicative of no dominant common factor. The mean PsyCap scores were 90.11 ± 9.18 for patients and 93.60 ± 12.13 for caregivers; the mean DC scores were 94.04 ± 14.31 and 97.68 ± 14.85, respectively. Paired-samples *t*-tests indicated that patients had significantly lower PsyCap and DC scores than caregivers (*p* < 0.05). Because the DCI was originally developed for spousal couples and adapted for non-spousal caregivers in this study, DCI total scores were additionally compared by relationship type. No significant differences were observed between spousal and non-spousal groups among either patients (92.93 ± 14.846 vs. 96.84 ± 12.519; *t* = −1.861, *p* = 0.064) or caregivers (96.58 ± 14.952 vs. 100.45 ± 14.317; *t* = −1.775, *p* = 0.077). Descriptive analysis showed that among PsyCap dimensions, patients scored lowest on resilience, followed by self-efficacy, whereas caregivers scored lowest on self-efficacy, followed by resilience. The difference in resilience between groups was significant (*p* < 0.05). Regarding DC dimensions, the lowest for patients was stress communication, followed by supportive coping, whereas for caregivers it was supportive coping, followed by stress communication. Except for stress communication, patients’ scores were lower than caregivers’ on all other DC dimensions, though these differences did not reach significance (*p* > 0.05). Detailed results are shown in Table 2.

**Table 2:** Comparison of psychological capital and dyadic coping scores between lung cancer patients and their caregivers.

Variables	Patients (*M* ± SD, *n* = 225)		Caregivers (*M* ± SD, *n* = 225)		Between-group comparison of total scores	
	Total score	Mean item score	Total score	Mean item score	*t*	*p*
PsyCap-Total	90.11 ± 9.18	3.47 ± 0.35	93.81 ± 11.83	3.60 ± 0.47	−4.443	<0.001**
Optimism	23.39 ± 3.91	3.90 ± 0.65	22.92 ± 5.07	3.82 ± 0.85	1.338	0.182
Hope	22.38 ± 3.34	3.73 ± 0.56	22.87 ± 3.58	3.81 ± 0.60	−1.496	0.136
Resilience	20.73 ± 2.89	2.96 ± 0.41	24.32 ± 4.31	3.48 ± 0.47	−12.792	<0.001**
Self-efficacy	23.60 ± 4.04	3.37 ± 0.58	23.72 ± 4.51	3.39 ± 0.64	−0.335	0.738
DC-Total	94.04 ± 14.31	2.69 ± 0.41	97.68 ± 14.85	2.79 ± 0.42	−4.380	<0.001**
Stress communication	9.75 ± 2.40	2.44 ± 0.60	9.44 ± 2.55	2.36 ± 0.64	1.713	0.088
Supportive dyadic coping	36.05 ± 8.31	2.57 ± 0.59	39.94 ± 9.67	2.85 ± 0.69	−5.474	<0.001**
Negative dyadic coping	23.94 ± 6.77	2.99 ± 0.85	25.79 ± 6.35	3.22 ± 0.79	−3.805	<0.001**
Delegated dyadic coping	10.56 ± 2.50	2.64 ± 0.63	9.83 ± 2.33	2.46 ± 0.58	3.648	<0.001**
Common dyadic coping	13.75 ± 3.25	2.75 ± 0.65	12.68 ± 3.48	2.54 ± 0.70	4.414	<0.001**

PsyCap, Psychological Capital; DC, Dyadic Coping, **p < 0.01.

### 3.3 Correlation between psychological capital and dyadic coping

Pearson correlation analysis revealed significant positive associations (*p* < 0.01) between patients’ and caregivers’ PsyCap and dyadic coping (see Table 3).

**Table 3:** Correlation analysis between psychological capital and dyadic coping among lung cancer patients and their caregivers.

Variables	PsyCap (patients)	PsyCap (caregivers)	DC (patients)	DC (caregivers)
PsyCap (patients)	1			
PsyCap (caregivers)	0.313**	1		
DC (patients)	0.214**	0.195**	1	
DC (caregivers)	0.198**	0.274**	0.636**	1

**p < 0.01.

### 3.4 Actor–partner interdependence model (APIM) analysis

We used patients’ and caregivers’ PsyCap as predictors and their dyadic coping as outcomes in an APIM-based SEM. Both actor effects were significant: patients’ PsyCap predicted their own coping (*β* = 0.17, *p* = 0.013, 95% CI [0.011, 0.497]) and caregivers’ PsyCap predicted their own coping (*β* = 0.23, *p* < 0.001, 95% CI [0.098, 0.372]). Regarding the partner effects, caregivers’ PsyCap significantly predicted patients’ dyadic coping (*β* = 0.14, *p* = 0.037, 95% CI [0.029, 0.306]), whereas the corresponding patient-to-caregiver partner effect was positive but did not reach statistical significance (*β* = 0.12, *p* = 0.065, 95% CI [−0.020, 0.497]). (Figure 1).

**Figure 1:**
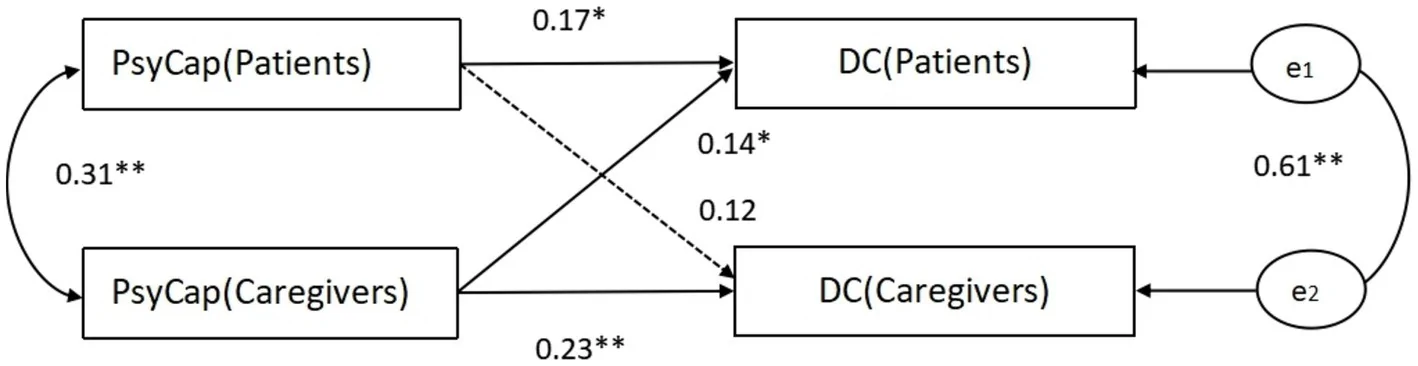
APIM path diagram of PsyCap and DC in lung cancer patients and caregivers. *p < 0.05. **p < 0.01. Solid lines indicate significant paths; Dashed lines indicate non-significant paths.

The APIM can follow various dyadic patterns: actor-only (*k* ≈ 0), partner-only (*k* → *∞*), mixed (*k* ≈ 0.5), dyadic (*k* ≈ 1), or contrast (*k* ≈ −1). Following the interchangeable and saturated (ISAT) approach described by Liu and Wu (2017), we tested whether patients’ and caregivers’ actor and partner effects could be constrained to equality. The equality-constrained model did not result in a statistically significant decrement in fit (*χ*^2^ = 0.347, *p* = 0.841), meeting the criterion of *p* > 0.20 recommended by Kenny and Ledermann (2010) for treating the actor and partner effects as statistically equivalent. This supported the use of the indistinguishable-dyad assumption and allowed estimation of the ratio of the partner effect to the actor effect (*k*). We found *k* = 0.633 (95% CI: 0.125–1.169), with the confidence interval encompassing both 0.5 and 1. We therefore tested models constraining *k* to 0.5 and 1. The *k* = 0.5 model yielded *χ*^2^ = 1.071, *χ*^2^/df = 0.268, CFI = 1.000, TLI = 1.027, *p* = 0.853, and RMSEA < 0.001. Because the unconstrained baseline APIM was saturated (df = 0), these global fit indices were not interpreted as evidence of superior absolute model fit. Rather, the *k* = 0.5 constraint was retained as a parsimonious representation of a mixed pattern of interdependence.

Given the high prevalence and clinical relevance of advanced cancer stage and comorbidity burden in the present sample, these two variables were additionally included as covariates in the APIM to examine the robustness of the observed associations. After adjustment, the four actor and partner effects remained consistent with the primary analysis: patients’ PsyCap significantly predicted their own dyadic coping (*β* = 0.16, *p* = 0.014) and caregivers’ dyadic coping (*β* = 0.13, *p* = 0.059), while caregivers’ PsyCap significantly predicted their own dyadic coping (*β* = 0.24, *p* < 0.001) and patients’ dyadic coping (*β* = 0.14, *p* = 0.029). Thus, the overall pattern and statistical significance of the associations remained unchanged after adjustment, supporting the robustness of the primary APIM findings. The corresponding covariate-adjusted APIM is presented in Supplementary Figure 1.

## 4 Discussion

### 4.1 Psychological capital among lung cancer patients and their caregivers

Our results showed that both lung cancer patients and their primary caregivers had lower levels of PsyCap compared to the general population, and even lower than those reported in breast cancer dyads (Zhang et al., 2024). The lowest dimensions were resilience and self-efficacy. This likely reflects lung cancer’s clinical profile—long treatment courses, heavy symptom burden, and poor prognosis—which can undermine patients’ confidence and positivity, leading to constrained PsyCap and social withdrawal (Varma et al., 2023; Wei et al., 2025).

Although caregivers’ overall PsyCap was higher than patients’, they still faced risk of psychological exhaustion. Long-term caregiving stress, fatigue, and anxiety can weaken caregivers’ self-efficacy and resilience. Additionally, over two-thirds of caregivers in this study juggled work and caregiving, and 67.11% of patients had other chronic diseases. Such role strain and financial pressures can further deplete caregivers’ psychological resources. Prior research shows caregivers’ PsyCap is closely linked to emotional exhaustion, caregiving satisfaction, and care quality; caregivers with higher PsyCap tend to experience lower burden and anticipatory grief (Barati Rizi and Pashaeypoor, 2024; Sun et al., 2024).

The four core components of PsyCap—hope, optimism, self-efficacy, and resilience—play protective roles in cancer adaptation. Hope and resilience help patients cope with uncertainty and stress; self-efficacy promotes active symptom management and decision-making; and optimism is associated with better adherence and prognosis. Higher levels of hope have been linked to lower depression, better coping, and post-traumatic growth; evidence for survival benefits remains inconclusive and requires further confirmation (Luthans and Youssef, 2004; Millstein et al., 2016; MacDonald et al., 2021; Thornton et al., 2021; Feldman and Corn, 2023). Accordingly, psychological capital interventions (PCI) are increasingly used to improve mental health and coping in chronic illness (Song et al., 2019). Evidence suggests PCI (e.g., web-based positive psychology programs, mindfulness, relaxation, and yoga) can significantly enhance mental wellbeing, quality of life, and emotional regulation (Han et al., 2023; Xin and Zhao, 2024).

Notably, PCI studies for cancer caregivers are limited. Existing research mainly targets other caregiving contexts, such as families of autistic children (Khedr et al., 2025). Given that caregivers’ PsyCap can indirectly affect patients’ recovery and quality of life through emotional transmission and communication, future research should evaluate PCI in cancer caregiving. Moreover, interventions should treat the patient–caregiver dyad as a unit to achieve mutual psychological growth.

### 4.2 Dyadic coping among lung cancer patients and their caregivers

Similar to PsyCap, the overall level of dyadic coping (DC) in lung cancer patients and caregivers was lower than that reported in other cancer populations, with patients scoring significantly lower than caregivers. In specific dimensions, patients scored lowest in stress communication, while caregivers scored lowest in supportive coping, indicating a weakness in emotional expression and responsiveness. Culturally, East Asian norms of endurance and “not burdening others” may lead patients to conceal distress, resulting in poor communication. Caregivers often feel a strong sense of duty, but chronic stress, sleep deprivation, and financial strain can deplete their emotional resources. This may lead to “over-rationalized support” or substituting instrumental assistance for emotional empathy (Kim and Schulz, 2008). As a result, caregivers may be efficient at practical tasks but less effective at providing emotional support, consistent with their lowest scores in supportive coping.

Lung cancer’s heavy burden and poor prognosis require sustained high-quality communication and collaboration between patient and caregiver throughout treatment and follow-up. Studies indicate that communication barriers are a core contributor to dyadic coping imbalance in high-stress health contexts (Falconier and Kuhn, 2019). Factors like physical suffering, financial stress, and uncertainty often prompt both partners to engage in protective buffering—suppressing or minimizing distress to avoid burdening the other. Although this may temporarily reduce anxiety, it undermines emotional synchrony and self-efficacy over time, weakening mutual understanding and overall coping capacity (Langer et al., 2009). Numerous studies confirm that open communication and active shared coping in cancer couples are associated with greater relationship satisfaction, emotional stability, and quality of life, whereas avoidant or protective patterns are linked to higher depression, anxiety, and relational tension. These findings underscore that “communication and support” are critical gaps in dyadic coping within lung cancer families (Badr and Taylor, 2006; Traa et al., 2015; van Roij et al., 2022).

Therefore, future interventions should emphasize communication skills and emotional support. Dyadic coping interventions developed for cancer couples have shown promising outcomes internationally. These programs (often grounded in psychoeducation and cognitive–behavioral therapy) aim to improve communication skills, foster emotional expression and empathy, and teach stress-management strategies that both partners apply collaboratively. Structured dyadic sessions involving goal setting, stress-dialogue practice, and emotion-regulation training have been shown to improve patients’ quality of life and psychological adjustment, while also reducing caregiver burden and enhancing relationship satisfaction and empathy (Chen et al., 2023; Liu et al., 2024). Developing culturally sensitive interventions to strengthen dyadic communication and supportive coping in lung cancer couples is therefore important. Since roughly 30% of caregivers in our study were non-spouses, such interventions should extend beyond spousal dyads to include non-spousal family caregivers as well.

### 4.3 Actor–partner effects between psychological capital and dyadic coping

A lung cancer diagnosis is a profound traumatic stressor for individuals and families. Earlier research often focused on the patient and partner support in a unidirectional model, neglecting systemic reciprocal effects within couples. Over the past two decades, scholars have shifted to a dyadic coping framework, recognizing that cancer as a shared stressor triggers mutual emotional resonance and interdependence between partners. Individuals’ psychological resources not only strengthen their own coping but also influence their partner’s coping through emotional co-regulation and behavioral modeling, creating a dynamic dyadic adjustment loop. According to Conservation of Resources (COR) theory, PsyCap is a personal resource that enhances stress resilience and can cross over to partners, promoting joint coping within the family system (Liu et al., 2021; Egozi Farkash et al., 2022).

Our results showed significant positive correlations between PsyCap and DC for both patients and caregivers. The APIM analysis indicated a mixed interdependence pattern, suggesting that each person’s coping is driven by both their own resources and their partner’s resources, reflecting partial interdependence and partial autonomy within the dyad. This supports the relational co-regulation hypothesis, emphasizing the bidirectional nature of emotional and behavioral exchange within couples facing stress (Kenny and Ledermann, 2010). Notably, the caregiver-to-patient partner effect was statistically significant, whereas the patient-to-caregiver partner effect was positive but did not reach statistical significance (*β* = 0.12, 95% CI [−0.020, 0.497], *p* = 0.065). Therefore, this finding should be interpreted cautiously and does not provide sufficient evidence for a definitive patient-to-caregiver partner effect. Although the relatively stronger caregiver-to-patient association may suggest a potential asymmetry in dyadic influence, this interpretation warrants further investigation. Similar findings were observed in prostate cancer dyads, where caregivers’ psychological resources significantly affected patients’ quality of life, while patient-to-caregiver partner effects were non-significant (Sauer et al., 2022). A plausible explanation is that lung cancer patients often focus inward on their symptoms and treatment, whereas caregivers undertake multiple responsibilities (daily care, financial support, emotional regulation) and thus play a more outward-facing, regulatory role. This asymmetry in influence may evolve over the disease trajectory, warranting longitudinal investigation.

Our study further indicates that both patients’ and caregivers’ PsyCap significantly predict their own dyadic coping. Moreover, caregivers with higher PsyCap tend to encourage patients’ engagement in illness management through positive communication and emotional feedback, whereas low caregiver PsyCap may transmit negative affect, amplifying patients’ feelings of helplessness and avoidance (Opsomer et al., 2022; McPherson and Devereaux, 2024). Therefore, dyad-oriented psychological support that involves both patients and caregivers may be worth considering in clinical settings; however, the effectiveness of PsyCap-focused dyadic programs for improving dyadic coping should be verified in longitudinal and randomized intervention studies (Chen et al., 2025; Wang et al., 2025). Importantly, the sensitivity analysis showed that the observed actor and partner effects remained stable after adjustment for cancer stage and comorbidity burden, suggesting that the primary APIM findingswere robust to these two specific clinical covariates.

### 4.4 Limitations

First, the cross-sectional design precludes causal inference or conclusions about directionality between PsyCap and dyadic coping. Second, convenience sampling from one tertiary hospital in one city may limit generalizability to other regions, care settings, and cultural contexts. Third, although Harman’s single-factor test did not indicate a dominant common factor, common method variance cannot be completely excluded because the study relied primarily on self-reported measures. Fourth, caregiver–patient relationships were heterogeneous (spouses vs. non-spousal family members), and the DCI wording was adapted for non-spousal caregivers. Formal measurement invariance across relationship types could not be tested due to insufficient subgroup sample sizes, and the observed lack of significant difference in total scores does not establish equivalence. Accordingly, cross-subgroup score comparability should be interpreted with caution, and future studies with larger samples are warranted. Furthermore, while total-scale Cronbach’s *α* coefficients were satisfactory for both instruments across both groups, a small number of subscales yielded values marginally below 0.70 (which may stem from their small number of items). Readers are advised against over-interpreting subscale-specific descriptive outcomes. Finally, although sensitivity analyses were conducted by adjusting for patient cancer stage and comorbidity burden, other potential confounders, such as treatment phase, symptom burden, pre-existing mental health conditions, and relationship quality, were not fully controlled. Future multi-center longitudinal research and randomized dyadic interventions with more comprehensive covariate adjustment and measurement-invariance testing are needed to confirm and extend these findings.

## 5 Conclusion

In summary, our APIM-based findings indicate that while PsyCap functions as an individual psychological resource, it also has reciprocal effects within the patient–caregiver dyad, with caregivers’ PsyCap playing a key role in facilitating patients’ coping. Accordingly, clinical practice and future interventions should adopt a positive psychology framework centered on the patient–caregiver dyad as a functional family unit. Interventions should aim to jointly enhance psychological capital, communication skills, and active coping strategies for both members, thereby fostering more effective mutual support and overall psychological adaptation.

## Data Availability

The raw data supporting the conclusions of this article will be made available by the authors, without undue reservation.
