# Correction: A study on the association between psychological capital and dyadic coping among lung cancer patients and their caregivers: an actor–partner interdependence model approach

**DOI:** 10.3389/fpsyg.2026.2014169

**Published:** 2026-10-22

**Authors:** Yan Li, Liu He, Vimala Ramoo, Xingling Zeng, Gek Mui Lim, Juan Du, Yuhong Zhang, Xiaoya Liu, Xinjing Xiang

**Affiliations:** 1 Faculty of Health Sciences, Nursing, and Education, MAHSA University, Jenjarom, Selangor, Malaysia; 2 The Second Affiliated Hospital of Chengdu Medical College, Nuclear Industry 416 Hospital, Chengdu, China; 3 Faculty of Medical and Life Sciences, Sunway University, Jalan University, Bandar Sunway, Selangor, Malaysia; 4 School of Nursing, Jiangsu Food ' Pharmaceutical Science College, Huai'an, Jiangsu, China

**Keywords:** actor–partner interdependence model, dyadic coping, lung cancer, patient–caregiver dyads, psychological capital, psycho-oncology

In the published article, the first paragraph of Section 2.1, “*Participants*,” incorrectly reported the recruitment period as January to June 2025 and the ethics approval number as “KY20232283-F-1.” The correct recruitment period is January to September 2025, and the correct ethics approval number is “YJ-2025-044-01.” In addition, as noted by the editor, the final sentence of this paragraph did not explicitly state whether written informed consent had been obtained.

A correction has been made to the section ***2.1 Participants***:

This study adopted a cross-sectional design and used convenience sampling. A total of 225 dyads of lung cancer patients and their primary caregivers were recruited from one tertiary hospital in Chengdu, Sichuan Province, China, between January and September 2025. This study was conducted in accordance with the Declaration of Helsinki and was approved by the Medical Ethics Committee of the Nuclear Industry 416 Hospital (Approval

No.: YJ-2025-044-01). Written informed consent to participate in this study was provided by the patients and their primary caregivers.

In addition, the first sentence of the **Methods** section in the Abstract has been corrected by replacing “June” with “September” to ensure consistency with the recruitment period reported in Section 2.1 “*Participants*.”

The original version of this article has been updated.

