# Supplementary material for: A study on the association between psychological capital and dyadic coping among lung cancer patients and their caregivers: an actor–partner interdependence model approach
Source: Front Psychol. 2026 Sep 17;17:1940535. doi: 10.3389/fpsyg.2026.1940535 (PMC13627295; doi:10.3389/fpsyg.2026.1940535)
Supplement: Supplementary file 1 [file Supplementary_file_1.docx]

Supplementary Material

## 1 Supplementary Figure

Supplementary Figure 1. Covariate-adjusted Actor-Partner Interdependence Model (APIM) controlling for patient cancer stage and comorbidity burden.

**Supplementary Figure 1.** **Covariate-adjusted Actor-Partner Interdependence Model (APIM) controlling for patient cancer stage and comorbidity burden.**

Notes: Standardized path coefficients are presented.**p* < 0.05. Solid lines indicate statistically significant paths (*p* < 0.05), and dashed lines indicate non-significant paths. The model was adjusted for patient cancer stage and comorbidity burden.
